# Establishing the Optimal Time for Induction of Labor in Women with Diet-Controlled Gestational Diabetes Mellitus: A Single-Center Observational Study

**DOI:** 10.3390/jcm11216410

**Published:** 2022-10-29

**Authors:** Patrik Šimják, Hana Krejčí, Markéta Hornová, Miloš Mráz, Antonín Pařízek, Michal Kršek, Martin Haluzík, Kateřina Anderlová

**Affiliations:** 1 Department of Gynecology and Obstetrics, 1st Faculty of Medicine, Charles University, General University Hospital in Prague, 128 08 Prague, Czech Republic; 2 3rd Department of Medicine, 1st Faculty of Medicine, Charles University, General University Hospital in Prague, 128 08 Prague, Czech Republic; 3 Diabetes Centre, Institute for Clinical and Experimental Medicine, 140 21 Prague, Czech Republic

**Keywords:** gestational diabetes mellitus, diet-control, labor induction, cesarean section, expectant management

## Abstract

To determine the optimal week for labor induction in women with diet-controlled gestational diabetes mellitus by comparing differences in perinatal and neonatal outcomes of labor induction to expectant management at different gestational weeks. **Methods**: This was a retrospective analysis of a prospectively recruited cohort of 797 singleton pregnancies complicated by diet-controlled gestational diabetes mellitus that were diagnosed, treated, and delivered after 37 weeks in a tertiary, university-affiliated perinatal center between January 2016 and December 2021. **Results**: The incidence of neonatal complications was highest when delivery occurred at 37 weeks, whereas fetal macrosomia occurred mostly at 41 weeks (20.7%); the frequency of large for gestational age infants did not differ between the groups. Conversely, the best neonatal outcomes were observed at 40 weeks due to the lowest number of neonates requiring phototherapy for neonatal jaundice (1.7%) and the smallest proportion of neonates experiencing composite adverse neonatal outcomes defined as neonatal hypoglycemia, phototherapy, clavicle fracture, or umbilical artery pH < 7.15 (10.4%). Compared with expectant management, the risk for neonatal hypoglycemia was increased for induction at 39 weeks (adjusted odds ratio 12.29, 95% confidence interval 1.35–111.75, *p* = 0.026) and that for fetal macrosomia was decreased for induction at 40 weeks (adjusted odds ratio 0.11, 95% confidence interval 0.01–0.92, *p* = 0.041), after adjusting for maternal pre-pregnancy body mass index, nulliparity, and mean pregnancy A1c. **Conclusions**: The lowest rate of neonatal complications was observed at 40 weeks. Labor induction at 40 weeks prevented fetal macrosomia.

## 1. Introduction

Gestational diabetes mellitus (GDM) is the most common metabolic complication in pregnancy; however, it remains a controversial topic. It is defined as glucose intolerance with onset or first recognition in the second or third trimester of pregnancy [1]. According to two recent meta-analyses, the prevalence in Europe ranges between 5.4–11.0% [2,3]. This wide range and the significant differences between regions are mainly due to the lack of international consensus regarding the optimal screening method and the cut-offs for the diagnosis of GDM [4]. After the release of the HAPO Study [5], the International Association of Diabetes in Pregnancy Study Groups (IADPSG) formulated recommendations for universal glucose tolerance testing in pregnancy [6]. Accordingly, every woman without overt glucose intolerance who had fasting glycemia <5.1 mmol/L in the first trimester undergoes standard 75 g 2 h fasting oral glucose tolerance test (OGTT) between 24–28 weeks of gestation. The cut-off glucose values for the diagnosis of GDM used were fasting plasma glucose ≥ 5.1 mmol/L or a plasma glucose ≥ 10.0 mmol/L or 8.5 mmol/L at 1 and 2 h, respectively. Women meeting these criteria for a diagnosis of GDM have been shown to have a significantly higher risk of perinatal complications, yet conclusive evidence confirming the efficacy of treatment is lacking. Thus, most clinicians in the United States use a two-step approach, first administering a 50 g non-fasting oral glucose challenge test at 24 to 28 weeks, followed by a 100 g fasting test for women who have a positive screening result [7]. The cut-off glucose values for the diagnosis of GDM are 5.3 mmol/L, 10.0 mmol/L, 8.6 mmol/L, and 7.8 mmol/L for fasting, 1, 2, and 3 h glycemia, respectively. For women diagnosed with GDM, the rate of certain perinatal complications was lower for the women randomized to treatment [8]. Alternatively, a plausible effect of treatment was also proven in women with GDM diagnosed through selective screening with a 75 g OGTT at 24 to 34 weeks who had fasting plasma glucose levels less than 7.8 mmol/L after an overnight fast and glycemia between 7.8 to 11.0 mmol/L at two hours [9]. Given this inconsistency of studies, many different guidelines were proposed for the diagnosis of GDM, which has to be considered when interpreting and comparing the results of different studies [10].

Following the diagnosis of GDM, women are recommended to initiate glucose monitoring, follow a low-carbohydrate diet, and partake in regular physical activity. In most cases, optimal glucose control is achieved with nutritional therapy and lifestyle modification alone (diet-controlled GDM or gestational diabetes type A1 (A1GDM)), with only 15–30% of women requiring therapy (medically controlled GDM or gestational diabetes type A2) [1]. GDM is an independent risk factor for fetal macrosomia, preeclampsia, primary cesarean section, and neonatal adiposity [11]. The inclusion of pharmacotherapy in the treatment of GDM is associated with an increased risk of cesarean section and preterm birth; however, metformin treatment likely reduces the incidence of macrosomia and neonatal hypoglycemia [12]. Diagnosis and optimal management of GDM improves perinatal outcomes by reducing the risk of preeclampsia (7.2% vs. 11.7%), macrosomia (birthweight > 4000 g; 8.4% vs. 17.4%), and shoulder dystocia (1.5% vs. 3.5%) [13]. The goal of GDM management is to minimize the incidence of maternal and fetal complications through tight glucose control and optimal mode and timing of delivery. In the case of pharmacologically treated GDM, guidelines [7,14] uniformly recommend induction of labor (IOL) at 39 weeks due to the higher risk of perinatal mortality and other complications [15,16]. In contrast, recommendations for mild forms of GDM (A1GDM) vary considerably. The American College of Obstetricians and Gynecologists (ACOG) guidelines recommend that women with diet-controlled GDM should deliver at 39 weeks of gestation [7], whereas the Society of Obstetricians and Gynecologists of Canada guidelines suggest IOL between 38 and 40 weeks depending on glycemic control and comorbidities [8]. Contrastingly, guidelines of the Czech Gynecological and Obstetrical Society do not recommend delivery prior to 40 weeks, with the possibility to delay induction of labor up to 41 weeks in selected cases. The rationale is to minimize iatrogenic interventions in otherwise low-risk pregnancies [17].

Given the lack of consensus among the different societies, we aimed to determine the optimal week for delivery in women with diet-controlled GDM diagnosed according to IADPSG criteria by comparing differences in perinatal and neonatal outcomes of labor induction at 39 and 40 weeks to expectant management.

## 2. Materials and Methods

### 2.1. Study Population

This was a retrospective analysis of a prospectively recruited consecutive cohort of singleton pregnancies complicated by GDM that were diagnosed, treated, and delivered in a single, tertiary, university-affiliated perinatal center between January 2016 and December 2021. Initially, 1008 Caucasian pregnant women with a positive screening for GDM were recruited between 24 and 28 weeks of gestation. Diagnostic criteria endorsed by International Association of Diabetes and Pregnancy Study Group (IADPSG) were applied [5]. The diagnosis was made if fasting plasma glucose ≥5.1 mmol/L twice at least 24 h apart or a plasma glucose ≥10.0 mmol/L or 8.5 mmol/L at 1 and 2 h, respectively, after a standardized 75 g-2 h oral glucose tolerance test (OGTT). The glucose solution was administered after the fasting glucose < 5.1 mmol/L was confirmed. The exclusion criteria were as follows: antenatal care or delivery at another facility, gestational age at delivery < 37 weeks, multiple pregnancy, and treatment with insulin or metformin. Ultimately, 797 women with diet-controlled GDM were eligible for analysis; they were stratified according to gestational age at delivery.

### 2.2. Antenatal Care

Women diagnosed with GDM received dietary counseling and underwent diabetologic surveillance at 4-week intervals by a diabetologist. A low-carbohydrate diet (carbohydrate intake limited to 200 g per day) and regular physical activity were recommended to all women. Glucose monitoring was self-performed five times a day using a personal glucometer, at least twice a week. Pharmacologic therapy was considered when, despite a diet and exercise, more than two blood glucose readings exceeded the target values of 5.3 mmol/L fasting or 7.8 mmol/L 1 h postprandial over 1 or 2 weeks. If GDM pharmacotherapy with metformin or insulin was initiated, the patient was excluded from the analysis. Antenatal obstetric care consisted of regular visits at 4-week intervals. According to CGOS recommendations, ultrasound biometry was performed at 36–38 weeks to exclude accelerated fetal growth. In women with diet-treated GDM, labor is not routinely induced before 40 weeks unless other risk factors are found. Indications for early induction included large-for-gestational-age (LGA) fetuses and other comorbidities.

### 2.3. Outcome Measures

Recorded data included maternal demographics such as age, body mass index (BMI) at conception, weight gain during pregnancy, parity, mode of conception, and chronic hypertension. Results of 75 g-OGTT and baseline and mean glycated hemoglobin (A1c) were also recorded. The primary outcome was the frequency of emergency cesarean sections, defined as cesarean sections performed after spontaneous onset of labor or IOL. Secondary perinatal and neonatal outcomes included preeclampsia (defined as gestational hypertension with at least one of the following new-onset conditions at or after 20 weeks: proteinuria (defined as an albumin to creatinine ratio >30 mg/mmol or loss of at least 300 mg of protein during a 24 h urine collection); acute kidney injury (creatinine ≥ 90 umol/L); liver involvement (elevated alanine transaminase or aspartate aminotransferase >40 IU/L); neurological complications; hematological complications (platelet count < 150,000/µL, disseminated intravascular coagulation, or hemolysis); uteroplacental dysfunction (fetal growth restriction, abnormal umbilical artery Doppler, or stillbirth) [18], intrahepatic cholestasis of pregnancy (pruritus during the second or third trimester of pregnancy with total bile acids >10 μmol/L in the absence of other causes of pruritus), macrosomia (birthweight > 4000 g), large for gestational age (LGA; birthweight > 90th centile for gestational age), SGA (birthweight < 10th centile for gestational age), umbilical artery pH, Apgar score at 5 min, neonatal hypoglycemia (glycemia < 2.5 mmol/L), jaundice requiring phototherapy, clavicle fracture, and neonatal intensive care unit (NICU) admission. Composite adverse neonatal outcomes (CANO) comprised neonatal hypoglycemia, phototherapy for jaundice, clavicle fracture, and umbilical artery pH < 7.15).

Perinatal and neonatal outcomes of women who underwent IOL at 39 and 40 weeks were compared with those of women who were managed expectantly. The expectant management group comprised women who delivered in the subsequent week following spontaneous onset of labor or IOL, which is in line with previously published approach [19].

### 2.4. Statistical Analysis

The study population was divided into five groups according to gestational age at the time of delivery. Continuous variables are presented as mean ± standard deviation or median and interquartile range. One-way ANOVA or Kruskal–Wallis test with multiple pairwise comparisons were used. Categorical variables are presented as percentages and numbers. Differences between the groups were compared using the chi-square test, and Fisher’s exact test was used to assess significance by group. Multivariate logistic regression analysis was performed to assess the impact of IOL on outcomes, after adjusting for pre-pregnancy BMI, nulliparity, and mean A1c. Statistical significance was assigned at *p* < 0.05. The analysis was performed using XLSTAT version 2020.5.1 (Addisonsoft, New York, NY, USA).

### 2.5. Power Analysis

The primary outcome was the frequency of emergency cesarean sections. Data of Gorgal et al. [20] reported the frequency of emergency cesarean sections in women with GDM as high as 19.5%. With the power (1-β) 0.8 and the number of estimated pairwise comparisons set to 4, the required sample size necessary to detect an effect of 10% is 406. As for the secondary outcomes, Kc et al. [21] reported the incidence of fetal macrosomia 15–45% among newborns of women with GDM. With the power (1-β) 0.8 and the number of estimated pairwise comparisons set to 4, the required sample size necessary to detect an effect of 6% is 693.

### 2.6. Ethical Approval

Written informed consent was obtained from each participant, and the study was approved by the Human Ethics Review Board of the General University Hospital in Prague, Czech Republic. This study was performed in accordance with the principles of the Declaration of Helsinki.

## 3. Results

The analysis included 797 women with diet-controlled GDM and singleton pregnancies who delivered at ≥37 weeks. After stratification using gestational age, no significant differences were observed in maternal age, pre-pregnancy BMI, and maternal weight gain during the pregnancy among the groups. There were fewer nulliparous women among those who delivered at 40 weeks than among those who delivered at 41 weeks. Conception after in vitro fertilization and chronic hypertension were more prevalent at earlier gestational ages (Table 1). Fasting 1- and 2 h glycemia values on the 75 g-OGTT were comparable between the groups; however, fewer women who delivered at 40 weeks were diagnosed using fasting glycemia. Additionally, no differences were observed between baseline and mean A1c values, suggesting that maternal glucose control was comparable between the groups and thus should not have influenced physician judgement when planning delivery (Table 1).

Labor onset and course differed according to gestational age (Table 2). Overall, elective cesarean sections accounted for 27.5% of all deliveries and majority were performed at 39 weeks, which is in accordance with local practice. Spontaneous onset of labor was recorded in 58.5% of women. The proportion was significantly lower at 41 weeks. Alternatively, frequency of IOL increased gradually with gestational age and reached 56.3% at 41 weeks. Overall, 14.1% of deliveries were induced. Emergency cesarean sections were performed in 14.5% of cases where vaginal delivery was intended. The total frequency of emergency cesarean sections following spontaneous onset of labor and IOL was significantly lower at 39 weeks and higher at 41 weeks, reaching 23.8%. This was mainly due to the significantly lower frequency of emergency cesarean sections in cases of spontaneous onset of delivery at 39 weeks.

A higher incidence of preeclampsia was noted in the group of women who delivered at 37 weeks, which justified the proactive approach at early term. Additionally, neonatal complications such as jaundice requiring phototherapy, neonatal hypoglycemia, and NICU admission were more common at 37 weeks. Accordingly, CANO was observed more frequently in this group (Table 3). In contrast, a significantly higher mean birthweight and incidence of fetal macrosomia were observed at 41 weeks, although the frequency of LGA did not differ between the groups (Table 3). A longer duration of pregnancy naturally extends the time during which the fetus can grow without increasing the growth velocity at the end of pregnancy. Conversely, the best neonatal outcomes were observed at 40 weeks, when the lowest number of neonates required phototherapy for neonatal jaundice and the proportion of neonates experiencing CANO was only 10.4% (Table 3).

**Figure 1:**
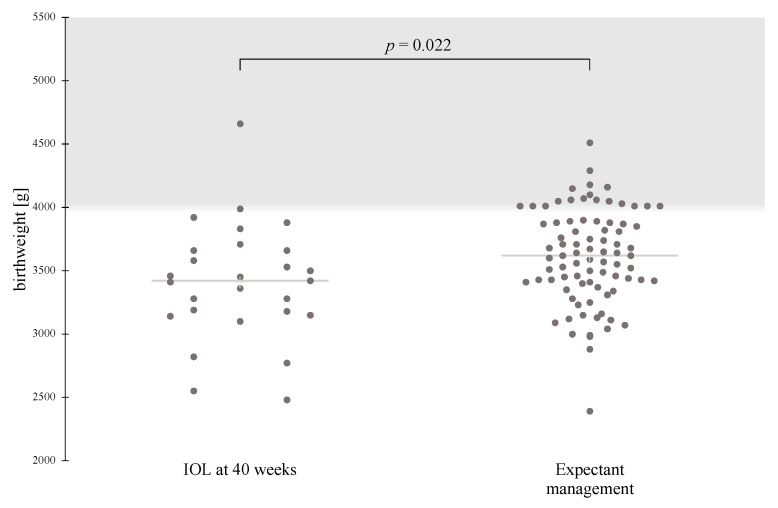
Scatterplot showing the mean birthweight and number of macrosomic newborns in the IOL at 40 weeks and Expectant management groups. Birthweight follows a normal distribution pattern, and data are compared using the *t*-test for the two independent samples. IOL, induction of labor.

When IOL was compared with expectant management, the risk for neonatal hypoglycemia was significantly increased for induction at 39 weeks, after adjusting for maternal pre-pregnancy BMI, nulliparity, and mean pregnancy A1c (adjusted odds ratio (aOR) 12.29, 95% CI 1.35–111.75, *p* = 0.026). Although the rate of emergency cesarean section following IOL at 39 weeks was higher compared with that following expectant management, this association was no longer statistically significant after adjusting for confounding factors (aOR 2.16, 95% CI 0.86–5.43, *p* = 0.102) (Table 4). Furthermore, the risk of fetal macrosomia was significantly lower in IOL at 40 weeks compared to expectant management (aOR 0.11, 95% CI 0.01–0.92, *p* = 0.041) (Table 5). Most of these cases of fetal macrosomia could be classified as grade 1 (4000 to 4499 g), with only one case of grade 2 (4500 to 4999 g) macrosomia in each group (Figure 1). The timing of IOL had no effect on pH < 7.15, NICU admissions, or clavicle fracture rates.

## 4. Discussion

In routine practice, obstetricians are often faced with the dilemma of deciding when to induce labor in a pregnant woman with mild, diet-controlled GDM so that the risk of fetal macrosomia, neonatal morbidity, and perinatal mortality is minimized without unnecessarily increasing the rates of cesarean sections. Additionally, a woman’s desire to avoid iatrogenic interventions may influence physician decision-making.

Our data suggest that the best time for IOL is 40 weeks, as the incidences of neonatal hypoglycemia and fetal macrosomia are lower at 40 weeks than at 39 and 41 weeks, respectively.

Neonatal hypoglycemia in women treated for GDM is usually mild and transient, but has been associated with poor neurodevelopmental outcomes and metabolic disturbances, including childhood obesity and type 2 diabetes [22]. In our study, the observed incidence of neonatal hypoglycemia was considerably lower than that in other published data [23], at 2.1%; owing to the exclusion of known risk factors such as preterm delivery and pharmacological treatment of GDM [24]. Furthermore, it should be emphasized that according to local protocol, the newborns of women with diet-treated GDM do not undergo routine glucose testing after birth. Indications for venipuncture or heel prick blood glucose testing included birthweight < 5th or >95th percentile or <2500 g, fetal asphyxia, and symptoms of hypoglycemia. The two cases of neonatal hypoglycemia detected in the group of women who underwent IOL at 39 weeks occurred in a newborn with low birthweight and in another who exhibited symptoms of hypoglycemia following emergency cesarean section for fetal hypoxia. There were five neonates with neonatal hypoglycemia in the expectant management group: two neonates with symptoms of hypoglycemia (one of them had impaired postnatal adaptation complicated by hypoxia (5 min Apgar score of 6)), two with low birthweight, and one with macrosomia. Routine screening could undoubtedly help in identifying additional cases of mild, otherwise asymptomatic hypoglycemia in both groups, affecting the results [25].

Further, IOL at 39 weeks may contribute to the observed trend towards a higher cesarean section rate. However, other studies do not support this finding. Several studies confirmed that IOL at term did not increase the risk of cesarean section in healthy pregnant women [26] or in women with GDM [19,27]. This observed trend could be partially explained by the decision to induce labor at 39 weeks due to associated pathologies such as hypertensive disorder, suspected SGA, and intrahepatic cholestasis of pregnancy. Contrastingly, in women with spontaneous onset of uterine activity, the rate of emergency cesarean sections was lowest at 39 weeks.

Feghali et al. discovered that the risk of emergency cesarean section in women with mild GDM following IOL was similar between 37 and 40 weeks, compared with expectant management [19]. The definition of expectant management and methodology were the same as in our study, and the basic maternal characteristics of the populations were comparable. In contrast, GDM was diagnosed using an ACOG-endorsed two-step approach: a 3 h 100 g OGTT was used for diagnosis, and IOL at 40 and 41 weeks were analyzed together as a single group. Additionally, secondary data analysis from a multi-center randomized controlled trial of mild GDM treatment demonstrated that compared with expectant management, IOL was not associated with increased cesarean section rates until 40 weeks [27]. However, in this study, mild GDM was considered as a mild elevation of glycemia during the 3 h 100 g OGTT regardless of the course and treatment of the disease in pregnancy. In our study, the trend towards an increased risk for emergency cesarean section following IOL at 39 weeks may have resulted from associated pathologies, such as ICP.

Additionally, our data demonstrated that IOL delayed to 41 weeks resulted in a significant increase in fetal macrosomia without increasing the frequency of LGA. Fetal macrosomia is a gestational age-dependent complication given the constant growth velocity of the fetus in the third trimester, in the absence of other pathology. It has been associated with severe neonatal and maternal complications including shoulder dystocia, clavicle fracture, brachial plexus injury, operative delivery, postpartum hemorrhage, and perineal trauma [21]. Early prenatal identification of fetuses expected to be macrosomic by the estimated date of delivery in women with GDM is key to improving obstetric outcomes, considering that IOL in these pregnancies is burdened with increased risks of emergency cesarean section, without improved perinatal outcomes [28]. Unfortunately, the clinician’s ability to accurately predict macrosomia using third-trimester ultrasound remains limited [29]. Allowing for an additional week of the constant growth of a mature fetus is an unnecessary risk, with no fetal or maternal benefit. Our study does not hate the power to assess the risk of perinatal death; however, a recent population-based cohort study showed that fetal macrosomia, regardless of underlying etiology, is a significant risk factor for intrauterine demise [30]. Furthermore, a French population-based study demonstrated that term pregnancies with diet-treated GDM were associated with a 30% increase in perinatal death due to delayed IOL, as it is believed to be a mild disorder [31]. Contrastingly, Karmon et al. found that the stillbirth rate after 40 weeks in women with diet-treated GDM was lower than that in normoglycemic women, which can be attributed to IOL at 40 weeks [32]. These arguments justify IOL at term for prevention of fetal macrosomia and perinatal death.

The strengths of our study lie in the prospective recruitment of women who underwent standardized GDM screening according to IADPSG recommendations and were followed up throughout pregnancy at a single clinic that provided both obstetric and diabetic care. A previously published methodology for determining the optimal time for IOL was used. The small sample size limited our ability to detect significant differences in less frequent complications. Additionally, we could not assess maternal outcomes, which may also affect the clinical management. This would have required a larger sample size and accurate diagnosis of maternal complications such as birth injuries by specialists at the time of delivery, which is beyond the capabilities of our clinic. Due to the low number of inductions prior to 39 weeks, we could not compare active and expectant management in the early term period; however, no current guideline supports IOL before 39 weeks in women with diet-treated GDM [7]. Understandably, our results are burdened with the risk of bias, as the decision to induce the labor is influenced by the knowledge of estimated fetal weight and maternal comorbidities. Only a randomized control trial can definitively answer the question of when it is optimal to induce the labor in women with diet-controlled GDM, but for ethical reasons it is unlikely that such a trial will be conducted soon. Therefore, we believe that observational studies can provide physicians with useful information for decision-making.

## 5. Conclusions

In our population of women with diet-controlled GDM, we observed the best neonatal outcomes at 40 weeks regardless of the mode of delivery. IOL at 39 weeks was inferior to expectant management as evidenced by the increased risk of neonatal hypoglycemia and unfavorable trend for higher rates of cesarean section following IOL. Delaying active management to 40 weeks improved neonatal outcomes that were comparable to those of expectant management. Additionally, induction at 40 weeks was associated with lower odds of fetal macrosomia. Thus, we concluded that in women with diet-treated GDM, the optimal time for labor induction is 40 weeks.

## Figures and Tables

**Table 1:** Pregnancy characteristics stratified by gestational age.

	37 wk	38 wk	39 wk	40 wk	41 wk	Total	*p*-Value
	*n* = 62	*n* = 136	*n* = 282	*n* = 230	*n* = 87	*n* = 797	
age [years]	34.0 ± 5.2	34.1 ± 5.2	33.7 ± 5.0	33.3 ± 5.3	33.0 ± 4.0	33.6 ± 5.1	0.436
BMI [kg/m^2^]	23.0 (20.4–27.0)	24.0 (21.0–29.0)	23.9 (21.0–27.0)	23.2 (20.8–27.0)	24.2 (21.0–28.8)	23.7 (21.0–27.5)	0.422
nulliparity	51.6 % (32)	58.1 % (79)	55.0.% (155)	46.5 % (107) *	67.8 % (59) *	54.2 % (432)	0.011
conception after IVF	17.7 % (11)	17.6 % (24) *	13.1 % (37)	7.8 % (18) *	8.0 % (7)	12.2 % (97)	0.023
chronic hypertension	9.7 % (6) *	5.1 % (7)	2.5 % (7)	3.0 % (7)	1.1% (1)	3.5 % (28)	0.033
weight gain to screening [kg]	7.0 (5.0–10.0)	8.0 (6.0–10.0)	7.5 (5.5–10.0)	7.0 (5.0–10.0)	7.6 (5.0–10.0)	7.5 (5.0–10.0)	0.988
weight gain in pregnancy [kg]	9.5 (6.5–14.0)	10.0 (7.0–14.0)	10.0 (7.0–13.8)	10.0 (7.0–13.0)	10.3 (6.8–13.0)	10.0 (7.0–13.3)	0.992
fasting plasma glucose	5.0 (4.6–5.4)	5.0 (4.5–5.4)	5.0 (4.6–5.3)	4.8 (4.5–5.3)	4.9 (4.5–5.3)	4.9 (4.5–5.3)	0.151
1 h plasma glucose [mmol/L]	10.3 (9.6–11.1)	10.3 (9.8–10.9)	10.3 (9.6–10.7)	10.2 (9.5–10.8)	10.2 (9.7–10.8)	10.3 (9.7–10.8)	0.981
2 h plasma glucose [mmol/L]	8.5 (6.9–9.1)	8.5 (7.2–8.9)	8.5 (7.3–9.0)	8.6 (7.0–9.1)	8.5 (7.4–8.9)	8.5 (7.2–9.0)	0.960
fasting glycemia ≥ 5.1 mmol/L	35.5 % (22)	39.0 % (53)	36.9 % (104)	27.4 % (63) *	35.6 % (31)	34.3 % (273)	0.129
positive 75 g OGTT	64.5 % (40)	61.0 % (83)	63.1 % (178)	72.6 % (167) *	64.4 % (56)	65.7 % (524)	
A1c at diagnosis [%; mmol/mol]	5.1 (4.8–5.4); 32.0 (29.3–35.0)	5.2 (4.9–5.4); 33.0 (30.0–35.0)	5.1 (4.8–5.3); 32.0 (29.0–34.0) †	5.1 (4.8–5.3); 32.0 (29.0–34.9)	5.1 (4.8–5.3); 32.0 (29.0–34.9)	5.1 (4.8–5.3); 32.0 (29.0–34.0)	0.244
mean A1c [%; mmol/mol]	5.2 (4.9–5.4); 33.0 (30.4–35.2)	5.2 (5.0–5.4); 33.0 (30.7–35.7)	5.2 (4.9–5.3); 32.5 (30.0–34.3) †	5.2 (4.9–5.4); 32.7 (30.0–35.0)	5.2 (4.9–5.4); 33.0 (30.3–35.0)	5.2 (4.9–5.4); 32.8 (30.0–35.0)	0.236

BMI, body mass index; IVF, in vitro fertilization; OGTT, 75 g-oral glucose tolerance test; A1c, glycated hemoglobin; wk, weeks Data are given as mean ± SD, *n* (%) or median (interquartile range). Continuous variables were compared using one-way ANOVA or Kruskal–Wallis test with multiple pairwise comparisons. Categorical variables were compared using chi-square and Fisher’s exact test to assess significance per cell. * *p* < 0.05 per cell by Fisher’s exact test, † *p* < 0.05 vs. 38 weeks.

**Table 2:** Delivery outcomes stratified by gestational age.

		37 wk	38 wk	39 wk	40 wk	41 wk	Total	*p*-Value
		*n*= 62	*n*= 136	*n*= 282	*n*= 230	*n*= 87	*n*= 797	
spontaneous onset		59.7 % (37)	65.4 % (89)	58.2 % (164)	61.3 % (141)	40.2 % (35) †	58.5 % (466)	<0.0001
	vaginal delivery ‡	81.1 % (30)	87.6 % (78)	95.1% (156) †	87.2 % (123)	85.3 % (29)	89.3 % (416)	0.029
	emergency CS ‡	18.9 % (7)	12.4 % (11)	4.9 % (8) †	12.8 % (18)	17.1 % (5)	10.7 % (50)	
labor induction		6.5 % (4)	5.1 % (7) †	8.9 % (25) †	11.7 % (27)	56.3 % (49) †	14.1 % (112)	<0.0001
	vaginal delivery §	75.0 % (3)	57.1 % (4)	64.0 % (16)	74.1 % (20)	71.4 % (35)	69.6 % (78)	0.866
	emergency CS §	25.0 % (1)	42.9 % (3)	36.0 % (9)	25.9 % (7)	28.6 % (14)	30.4 % (34)	
elective CS		33.9 % (21)	29.4 % (40)	33.0 % (93) *	27.0 % (62)	3.4 % (3) †	27.5 % (219)	<0.0001
emergency CS ¶		19.5 % (8)	14.6 % (14)	9.0 % (17) †	14.9 % (25)	23.8 % (20) †	14.5 % (84)	0.023

CS, cesarean section; wk, weeks; Data are presented as percentages and numbers; they were compared using chi-square and Fisher’s exact test to assess significance per cell. * *p* < 0.05 per cell by Fisher’s exact test; † *p* < 0.01 per cell by Fisher’s exact test; ‡ % from the spontaneous onset group; § % from the induction of labor group; ¶ % from the spontaneous onset and labor induction.

**Table 3:** Perinatal and neonatal outcomes stratified by gestational age.

	37 wk	38 wk	39 wk	40 wk	41 wk	Total	*p*-Value
	*N* = 62	*n*= 136	*n*= 282	*n*= 230	*n*= 87	*n*= 797	
preeclampsia	6.5 % (4) *	2.9 % (4)	1.4 % (4)	1.3 % (3)	0	1.9 % (15)	0.036
ICP	4.8 % (3)	8.1 % (11)	6.7 % (19)	2.6 % (6)	0 *	4.9 % (39)	0.017
birthweight [g]	2992 ± 412 †	3233 ± 413	3311 ± 388	3457 ± 398 †	3595 ± 388 †	3346 ± 424	<0.0001
macrosomia	1.6 % (1)	2.9 % (4)	4.6 % (13)	7.0 % (16)	20.7 %(18) †	6.5 % (52)	<0.0001
LGA	16.1% (10)	18.4 % (25)	11.3 % (32)	16.1 % (37)	12.6 % (11)	14.4 % (115)	0.269
SGA	4.8 % (3)	1.5 % (2)	3.2 % (9)	3.9 % (9)	6.9 % (6)	3.6 % (29)	0.295
phototherapy	21% (13) †	5.1% (7)	5.7 % (16)	1.7 % (4) †	6.9 % (6)	5.8 % (46)	<0.0001
NICU admission	9.7 % (6) †	2.9 % (4)	2.1 % (6)	1.3 % (3)	2.3 % (2)	2.6 % (21)	0.007
5 min APG < 7	0	0.7 % (1)	0	0	1.1 % (1)	0.3 % (2)	0.238
umbilical artery pH < 7.15	5.9 % (3)	9.6 % (11)	8.1 % (18)	6.8 % (11)	7.9 % (6)	7.9 % (49)	0.900
neonatal hypoglycemia	6.5 % (4) *	1.5 % (2)	2.1 % (6)	1.7 % (4)	1.2 % (1)	2.1 % (17)	0.171
clavicle fracture ‡	3.0 % (1)	2.4 % (2)	3.5 % (6)	3.5 % (5)	4.7 % (3)	3.4% (17)	0.967
CANO	29.0 % (18) †	15.4 % (21)	15.6 % (44)	10.4 % (24) *	16.1 % (14)	15.2 % (121)	0.010

wk, weeks; ICP, intrahepatic cholestasis of pregnancy; LGA, large for gestational age; SGA, small for gestational age; NICU, neonatal intensive care unit; APG, Apgar; CANO, composite adverse neonatal outcome (neonatal hypoglycemia, phototherapy, clavicle fracture, umbilical artery pH < 7.15). Continuous variables were compared using one-way ANOVA or Kruskal–Wallis test with multiple pairwise comparisons Data are presented as percentages and numbers; they were compared using the chi-square and Fisher’s exact tests to assess significance per cell. * *p* < 0.05 per cell by Fisher’s exact test; † *p* < 0.01 per cell by Fisher’s exact test. ‡ the proportion of vaginal births.

**Table 4:** Induction of labor at 39 weeks vs. Expectant management.

Outcome	IOL at 39 Weeks *n* = 25	Expectant Management *n* = 252	aOR (95% CI)	*p*-Value
emergency cesarean section	36.0 % (9)	17.9 % (45)	2.16 (0.86–5.43)	0.102
macrosomia	0	11.5 % (29)	NA	NA
LGA	4.0 % (1)	13.9 % (35)	0.33 (0.04–2.55)	0.287
SGA	4.0 % (1)	4.8 % (12)	0.76 (0.09–6.21)	0.799
5 min APG < 7	0	0.4 % (1)	NA	NA
Phototherapy	4.0 % (1)	3.2 % (8)	1.04 (0.12–8.87)	0.973
NICU	4.0 % (1)	2.0 % (5)	3.30 (0.31–35.29)	0.324
umbilical artery pH < 7.15 *	12.5 % (3)	7.3 % (16)	2.05 (0.53–7.87)	0.296
neonatal hypoglycemia	8.0 % (2)	2.0 % (5)	12.29 (1.35–111.75)	0.026
clavicle fracture	0	3.2 % (8)	NA	NA
CANO	24.0 % (6)	13.9 % (35)	2.21 (0.80–6.15)	0.127

IOL—induction of labor; LGA, large for gestational age; SGA, small for gestational age; NICU, neonatal intensive care unit; APG, Apgar; CANO, composite adverse neonatal outcome (neonatal hypoglycemia; phototherapy, clavicle fracture, umbilical artery pH < 7.15); CI, confidence interval; aOR, adjusted odds ratio. Multivariate logistic regression, adjusted for pre-pregnancy BMI, nulliparity, and mean A1c. * 12.6 % missing data.

**Table 5:** Induction of labor at 40 weeks vs. Expectant management.

Outcome	IOL at 40 Weeks *n* = 27	Expectant Management *n* = 84	aOR (95% CI)	*p*-Value
emergency cesarean section	25.9 % (7)	23.8 % (20)	1.36 (0.45–4.11)	0.586
macrosomia	3.7 % (1)	21.4 % (18)	0.11 (0.01–0.92)	0.041
LGA	18.5 % (5)	13.1 % (11)	1.27 (0.38–4.27)	0.704
SGA	14.8 % (4)	6.0 % (5)	4.44 (0.94–20.90)	0.059
5 min APG < 7	0	1.2 % (1)	NA	NA
Phototherapy	0	6.0 % (5)	NA	NA
NICU	3.7 % (1)	2.4 % (2)	1.00 (0.03–32.74)	1.000
pH < 7.15 *	14.8 % (4)	8.0% (6)	1.87 (0.42–8.44)	0.415
neonatal hypoglycemia	3.7 % (1)	1.2 % (1)	4.31 (0.18–102.97)	0.367
clavicle fracture	3.7 % (1)	3.6 % (3)	1.16 (0.10–14.27)	0.909
CANO	22.2 % (6)	15.5 % (13)	1.33 (0.39–4.60)	0.649

IOL, induction of labor; LGA, large for gestational age; SGA, small for gestational age; NICU, neonatal intensive care unit; APG, Apgar; CANO, composite adverse neonatal outcome (neonatal hypoglycemia; phototherapy, clavicle fracture, umbilical artery pH < 7.15); CI, confidence interval; aOR, adjusted odds ratio. Multivariate logistic regression, adjusted for pre-pregnancy BMI, nulliparity, and mean A1c. * 10.8 % missing data.

## Data Availability

The data that support the findings of this study are available on re- quest from the corresponding author.
